# Multi-Omics Reveals That the Rumen Transcriptome, Microbiome, and Its Metabolome Co-regulate Cold Season Adaptability of Tibetan Sheep

**DOI:** 10.3389/fmicb.2022.859601

**Published:** 2022-04-13

**Authors:** Xiu Liu, Yuzhu Sha, Weibing Lv, Guizhong Cao, Xinyu Guo, Xiaoning Pu, Jiqing Wang, Shaobin Li, Jiang Hu, Yuzhu Luo

**Affiliations:** ^1^Gansu Key Laboratory of Herbivorous Animal Biotechnology, College of Animal Science and Technology, Gansu Agricultural University, Lanzhou, China; ^2^Animal Husbandry and Veterinary Station in Huangyuan County, Xining, China

**Keywords:** Tibetan sheep, rumen, transcriptome, microorganism, metabolome, cold season

## Abstract

Tibetan sheep can maintain a normal life and reproduce in harsh environments under extreme cold and lack of nutrition. However, the molecular and metabolic mechanisms underlying the adaptability of Tibetan sheep during the cold season are still unclear. Hence, we conducted a comprehensive analysis of rumen epithelial morphology, epithelial transcriptomics, microbiology and metabolomics in a Tibetan sheep model. The results showed that morphological structure of rumen epithelium of Tibetan sheep in cold season had adaptive changes. Transcriptomics analysis showed that the differential genes were primarily enriched in the PPAR signaling pathway (ko03320), legionellosis (ko05134), phagosome (ko04145), arginine and proline metabolism (ko00330), and metabolism of xenobiotics by cytochrome P450 (ko00980). Unique differential metabolites were identified in cold season, such as cynaroside A, sanguisorbin B and tryptophyl-valine, which were mainly enriched in arachidonic acid metabolism, arachidonic acid metabolism and linolenic acid metabolism pathways, and had certain correlation with microorganisms. Integrated transcriptome-metabolome-microbiome analysis showed that epithelial gene-*GSTM3* expression was upregulated in the metabolism of xenobiotics by the cytochrome P450 pathway during the cold season, leading to the downregulation of some harmful metabolites; *TLR5* gene expression was upregulated and *CD14* gene expression was downregulated in the legionellosis pathway during the cold season. This study comprehensively described the interaction mechanism between the rumen host and microbes and their metabolites in grazing Tibetan sheep during the cold season. Rumen epithelial genes, microbiota and metabolites act together in some key pathways related to cold season adaptation.

## Introduction

Tibetan sheep are primitive sheep bred under special environmental conditions on the Qinghai-Tibet Plateau (high cold, low oxygen and strong ultraviolet rays). Grazing Tibetan sheep are widely affected by plateau ecological factors such as cold and nutritional stress during the cold season. The alpine grassland area of the Qinghai-Tibet Plateau can be divided into warm and cold seasons, corresponding to the grassy and the withed stage of grassland vegetation change, respectively. During the year, Tibetan sheep primarily obtain nutrients by eating natural forages, while during the long withered season (cold season), Tibetan sheep can only obtain nutrients by eating withered grass. The nutritional supply of Tibetan sheep during the warm and cold seasons is severely unbalanced, and they are under more severe nutritional stress during the cold season, leading them to form a growth pattern of “fat loss in cold season and rejuvenation in warm season.” Their production efficiency is low, which seriously limits the utilization of germplasm characteristics and industrial development of Tibetan sheep.

Food digestion and absorption is a key process of animal adaptive evolution. In addition to the role of the animal’s own genome, the intestinal symbiotic microbiome also plays an important role in helping the host digest food, synthesizing nutrients that cannot be synthesized by the host itself, thus expanding the animal’s metabolic reservoir (Hess et al., 2011; Muegge et al., 2011). The host and microbial genomes must coordinate their work and perform their respective functions to maintain their body health under various environmental conditions (Allan and Stankey, 2009). In addition, some human-based cross-sectional studies have identified external and internal effects, such as diet, environment, drugs, and host immune and metabolic status, but these effects account for only 10–20% of the microbial diversity (Zhernakova et al., 2016; Kurilshikov et al., 2017), and the diversity of most microorganisms among individuals is unexplainable. Therefore, more attention has been given to the integration of the host genome, transcriptome, epigenome, metabolome, and microbiome interactions.

An increasing number of studies have shown the importance of uncovering the relevant laws and mechanisms underlying host physiological characterization by sequencing the gut microbiome and metagenomics comprehensively and correlating these data with the host genome, transcriptome and metabolic profile (Wang Q. et al., 2019). Based on multi-omics studies, the rumen microbiome and its metabolome and host metabolome have been shown to promote the individualized production performance of dairy cows jointly (Xue et al., 2020); a comprehensive transcript and microbiome analysis showed that nutritional intervention improved the rumen function of stunted yaks and promoted compensatory growth (Hu et al., 2019). The interactions among the calf rumen microbiome, rumen epithelial transcriptome, and microbial metabolites suggest that a highly active early microbiome regulates rumen development at the cellular level, and miRNAs may mediate these host-microbial interactions (Malmuthuge et al., 2019). At present, there have been many reports revealing the microbiota composition and gene function of the digestive tracts of ruminants living on the Qinghai-Tibet Plateau (Zhang et al., 2016), but there is still a lack of information on rumen microbiota and the interaction of metabolites with the host in Tibetan sheep. We measured the rumen flora and rumen fermentation parameters of the grazing Tibetan sheep in the warm (July) and cold (December) seasons and found that the rumen SCFAs (Short chain fatty acids) content and microbial flora abundance of Tibetan sheep in the cold season were significantly higher than those in the warm season; and interaction analysis showed that rumen microbial-SCFA-host genes had a certain correlation (Liu et al., 2020), and the interaction between microbial flora density and SCFAs may be related to its adaptability to warm and cold seasons.

Therefore, we hypothesized that the rumen microbiome and metabolome actively participate in Tibetan sheep plateau adaptability during the warm and cold seasons through interactions with the host transcriptome. We used integrated bioinformatics-based 16S rRNA sequencing, a metabolome determination of rumen microbes and rumen transcriptome (mRNA sequencing) to explore the interaction between the host and microbes and their regulatory role in plateau adaptation during the cold and warm seasons.

## Materials and Methods

### Ethics Statement

All studies involving animal were carried out in accordance with the regulations for the Administration of Affairs Concerning Experimental Animal (Ministry of Science and Technology, China; revise in June 2004), and sample collection protocols were approved by the Livestock Care Committee of Gansu Agricultural University (Approval No. GAU-LC-2020-27).

### Experimental Design and Sample Collection

**FIGURE 1:**
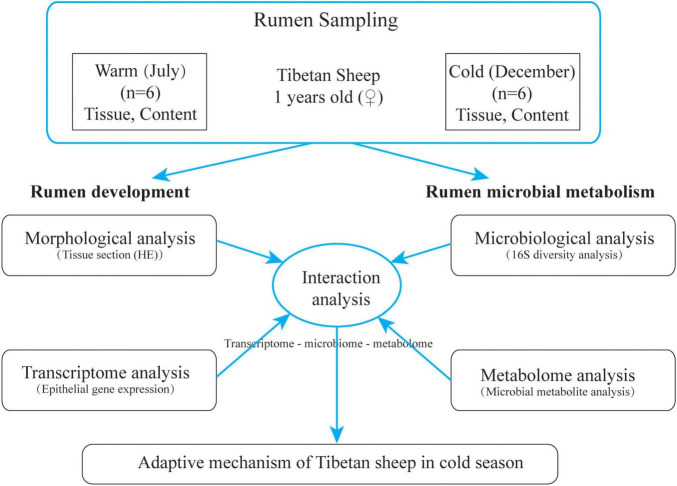
Schematic diagram of test design.

The grazing Tibetan sheep in Zuogaimanma Township, Hezuo City, Gannan Tibetan Autonomous Prefecture, Gansu Province, were taken as the research object, with an altitude of 3,300 m. Twelve Tibetan ewes (1 year old) with similar weight (35.12 ± 1.43 kg) and good health conditions were randomly selected from the same herder’s flock, all of which were in the local traditional natural grazing management state without any supplementary feeding. Samples were collected in the warm season (July) of 2019 and the cold season (December) of 2019. Before grazing in the morning, the rumen fluid was collected by sheep using gastric tube rumen sampler. The collected rumen fluid was quickly frozen in liquid nitrogen tank and brought back to the laboratory for storage at −80°*C* for subsequent 16S rRNA analysis and metabolite identification. After slaughter, rumen ventral sac tissue (1 cm^2^) was collected and fixed with 4% paraformaldehyde for morphological analysis; at the same time, rumen ventral bursa tissue was cut and quickly rinsed with PBS, then epithelial tissue was separated and quickly stored in liquid nitrogen for subsequent extraction of total RNA. The experimental design is shown in Figure 1.

### Morphological Characteristics

The rumen ventral sac tissue was fixed in 4% paraformaldehyde for 24 h, and then dehydration, transparency, waxing, embedding, slicing, and dyeing. Hematoxylin and eosin were used for staining. Hematoxylin stained the nucleus blue-purple, and eosin stained the cytoplasm pink. The thickness of rumen muscle layer, papillary height, papillary width, cuticle thickness, granular layer thickness, spinous layer thickness, and basal layer thickness were determined using CaseViewer section analysis system.

### RNA Extraction, Transcriptome Analysis, and Reverse Transcription-Quantitative PCR Verification

Trizol reagent method (DP762-T1C) was used to extract total RNA from the rumen epithelial tissue of Tibetan sheep, Nanodrop2000 was used for concentration detection. Agient2100, LabChip GX (platinum, Model Platinum Elmer LabChip GX, United States) for integrity testing. The cDNA Library was constructed by the VAHTS Universal V6 RNA-SEQ Library Prep Kit for Illumina^®^ Kit (NR604-02), and the Kit procedure was strictly followed. The products were further purified using a VAHTSTM DNA Clean Beads kit (N411–03). An Illumina NovaseQ6000 (San Diego) was used for the machine sequencing of the constructed library. A bioinformatics analysis was performed on the Biomark cloud platform BMKCloud,^1^ and clean data were obtained after data filtering from raw data. Clean data were sequenced with the specified reference genome Ovis_aries (Oar_rambouillet_v1.0. Ovis_aries) by HISAT (Li et al., 2015), and mapped data were obtained. StringTie (Kim et al., 2015) was used to compare reads on the pair for assembly. FPKM (Trapnell et al., 2010) (fragments per kilobase of transcript per million fragments mapped) was used to measure the transcription or gene expression level. The DESeq2 (Love et al., 2014) data analysis method was used to analyze differentially expressed genes [the fold change (FC) represents the ratio of expression levels between two samples (groups), the FC was obtained by adjusting the *p* value of significance], using fold change ≥ 2 and FDR < 0.01 as the screening criteria for differentially expressed genes; and the Gene Ontology (GO) and Kyoto Encyclopedia of Genes and Genomes (KEGG) functional enrichment analyses of differential genes were performed by GOseq (Young et al., 2010). To verify the reproducibility and reproducibility of the gene expression data, the RNA-seq method was used to select eight DEGs (Differentially expressed gene) randomly from the individual RNA samples originally extracted by RNA-seq for reverse transcription-quantitative PCR (RT–qPCR). See Supplementary Table 1 for the primer information for these DEGs.

### Microbial Diversity Analysis and LS-MS/MS Metabolic Profile Determination

Microbial DNA extraction, high-throughput sequencing, and bioinformatics analysis have been described in previous reports (Liu et al., 2020). Microbial species at the genus level were selected for the correlation analysis of this study using16S rRNA sequencing results.

Twelve rumen samples were analyzed with a liquid chromatography-mass spectrometry platform. After the sample was thawed at room temperature, 100 μL of sample was weighed each time, 500 μL of extract containing internal standard (1,000:2) (volume ratio of methanol to acetonitrile = 1:1, internal standard concentration 2 mg/L) was added, vortex mixed for 30 s, and then an ice water bath was followed by ultrasound for 10 mins, followed by standing at −20°C for 1 h, and centrifuging at 4°C for 15 mins (12,000 rpm). Then, 500 μL of supernatant was dispensed into an EP tube, and the extract was dried in a vacuum concentrator. A 150 μL extract (acetonitrile water volume ratio: 1:1) was added to the dried metabolite for resolution, followed by 30 s of vortexing, 10 min of ice water bath ultrasound, and 15 min of centrifugation at 4°C (12,000 rpm). Lastly, 120 μL of the supernatant was removed into a 2 mL injection bottle, and 10 μL of each sample was mixed to form a QC sample for computer testing. The LC/MS system for metabolomics analysis was composed of a Waters Acquity I-Class PLUS Ultra High Performance Liquid Tandem Waters Xevo G2-XS QToF High Resolution Mass Spectrometer, and the column used here was purchased from a Waters Acquity UPLC HSS T3 column (1.8 μm 2.1 × 100 mm). The samples were eluted using positive (ESI+) and negative (ESI−) mobile phases consisting of water and 5% acetonitrile, 0.1% formic acid as solvent A and acetonitrile and 0.1% formic acid as solvent B at a flow rate of 0.35 mL/min and 400 μl/min. The subsequent mobile phase (A:B) elution gradient was 0–0.25 min 98–2%, 10.0–13.0 min 2–98%, and 13.1–15.0 min 98–2%, followed by ion source temperature: 150°C and desolvation temperature: 500°C. The flow rates of the backblow and desolvent gas were 50 and 800 L/h, respectively. The original data collected by MassLynx V4.2 were used for peak extraction, peak alignment and other data processing operations by Progenesis QI software. Based on the Progenesis QI software, online databases such as METLIN and self-built databases of BMG were used for metabolite identification according to sample types. The mass number deviation of fragment ion recognition is less than 100 PPM. BMKCloud (see footnote text 1) was used to conduct a subsequent bioinformatics analysis on the identified metabolites. The differential metabolites were screened by combining the differential multiple, *P* value of the t-test and VIP value of the OPLS-DA model, and the screening standard was FC > 1, *P* value < 0.05 and VIP > 1, and KEGG functional annotation and enrichment analysis were performed for differential metabolites.

### Data Analysis

The independent sample T test in the IBM SPSS Statistics 25 software was used for the statistical analysis of the morphological data, and the difference was significant and statistically significant with *P* < 0.05; Spearman correlation test was used to analyze the correlation between genus-level rumen microbes (Top20) and differential metabolites and differential genes.

## Results

### Morphological Analysis of Rumen Epithelium

**FIGURE 2:**
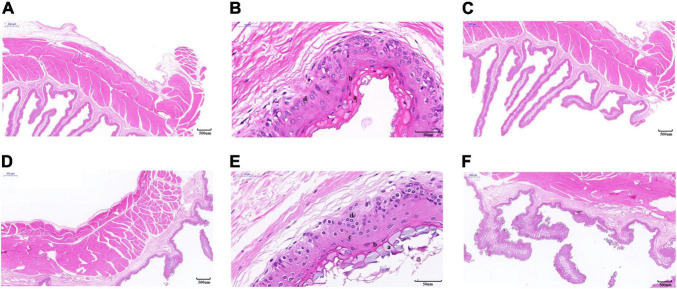
Morphology of rumen epithelial tissue of Tibetan sheep in cold and warm seasons. **(A–C)** Warm season, **(D–F)** cold season; **(a)** Cuticle, **(b)** Granular layer, **(c)** Spinous layer, **(d)** Basal layer.

As shown in Table 1, the thickness of the muscular layer of the rumen wall was significantly greater during the cold season than it was in the warm season (*P* < 0.01) (Figures 2A,D), the nipple width of the rumen epithelial layer was significantly greater in the cold season than in the warm season (*P* < 0.05), and the nipple height in the warm season was significantly greater than that in the cold season (*P* < 0.05) (Figures 2C,F). The thicknesses of the stratum corneum, granular layer, and spinous layer were significantly higher in the cold season than in the warm season (*P* < 0.01) (Figures 2B,E), and the thickness of the basal layer in the warm season was significantly higher than that in the cold season (*P* < 0.01). Figure 2 shows that the thickness of the musculature during the cold season is significantly higher than that in the warm season (*P* < 0.01); The stratum corneum in the rumen epithelial layer during the cold season shows signs of partial shedding (Figure 2A), and the shedding degree is greater than that in the warm season; in addition, the gap between the rumen papilla during the cold season is larger than that in the warm season, and the rumen papilla falls off to a greater degree.

**TABLE 1:** Morphological analysis of rumen epithelium in cold and warm seasons.

project/um	Warm season	Cold season	*P* Value
Muscle thickness	1,558.76 ± 20.62	1,961.5 ± 49.43	<0.001
Nipple height	3,333.82 ± 126.0	2,881.44 ± 94.49	0.021
Nipple width	318.7 ± 14.09	600.86 ± 23.15	<0.001
Stratum corneum thickness	35.72 ± 0.91	47.82 ± 1.34	<0.001
Granular layer thickness	10.9 ± 0.58	15.96 ± 0.61	<0.001
Spinous layer thickness	21.42 ± 0.84	40.18 ± 0.86	<0.001
Base layer thickness	24.58 ± 0.47	22.16 ± 0.49	0.007

### Rumen Epithelial Transcriptome (RNA-Seq) Analysis

#### Analysis of Differentially Expressed Genes

**FIGURE 3:**
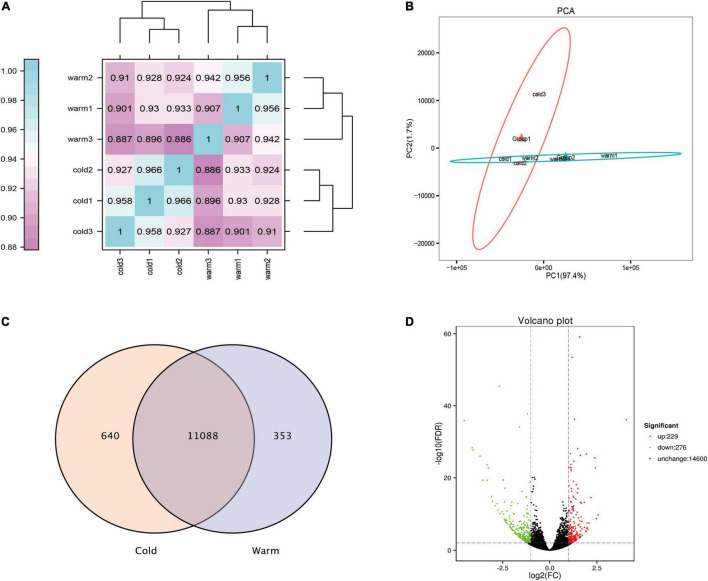
Analysis of rumen epithelial transcriptome differences in cold and warm seasons. **(A)** Heat map analysis of correlation between samples. **(B)** Principal component analysis of total gene expression in rumen epithelium during cold and warm seasons. **(C)** Venn diagram analysis of all expressed genes in rumen epithelium during cold and warm seasons. **(D)** Volcanic map of gene expression in rumen epithelium during cold and warm seasons.

In this study, transcriptome sequencing analysis was performed on the rumen epithelial tissue of Tibetan sheep during cold and warm seasons, and 101.42 Gb clean data were obtained. The clean data from each sample reached 15.56 Gb, and the percentage of Q30 bases was 94.79% and above. The correlation diagram shows that the correlation values within the warm group were all greater than 0.907, and the correlation values within the cold group were all greater than 0.927 (Figure 3A). PCA showed that there was a significant difference between the warm group and the cold group, and the repeatability within each group was good (Figure 3B). During the cold and warm seasons, 11,728 and 11,441 expressed genes were detected, of which 11,088 were co-expressed genes (Figure 3C). Using fold change ≥ 2 and FDR < 0.01 as the screening criteria, a total of 505 differentially expressed genes were found during the cold and warm seasons, of which 229 were upregulated during the cold season and 276 were downregulated (Figure 3D). Compared with the warm season, the five genes with the highest expression in the cold season were *TCHH*, *LOC114118453*, *GSTM3*, *DECR1*, and *LOC101106610*. Their expression reached 2.6, 3.0, 2.0, 2.4, and 2.0 times the expression in the warm season, respectively. The five genes with the lowest expression in the cold season were *KLK8*, *MUC13*, *CLCA4*, *SI*, and *TMC5*. In addition, in the cold season upregulated genes, the genes with FC > 4 and FPKM > 1 were screened: *LOC101113965*, *LOC114113812*, *LOC114117870*, *newGene_6417*, *LIX1*, *ERC2*, *B4GALNT2*, *CDH7* (FC > 4, FPKM > 1), where *LOC101113965* increased significantly, by 16 times (Supplementary Material 1).

#### Functional Enrichment Analysis of Differentially Expressed Genes

**FIGURE 4:**
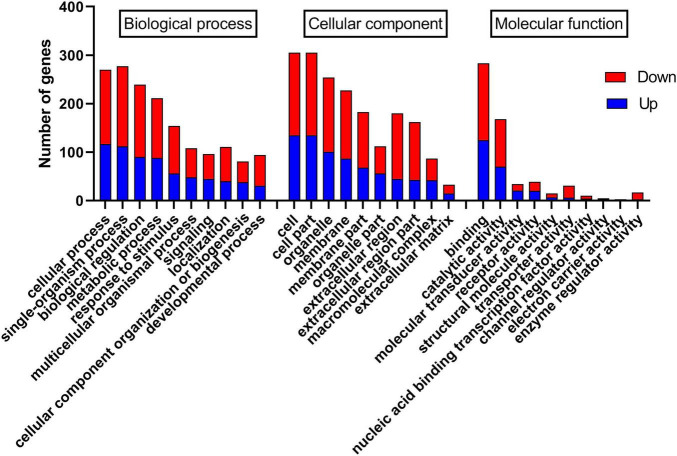
Gene ontology (GO) classification map.

The differentially expressed genes were annotated, and 420 and 327 differentially expressed genes were annotated in the GO and KEGG databases, respectively. In the GO database, annotated genes are divided into three categories: biological processes (BP), cell components (CC), and molecular functions (MF). As shown in Figure 4, GO functional classification found that most genes were enriched in cells (GO:0005623), cell part (GO:0044464), organelle (GO:0043226), membrane (GO:0016020), and membrane part (GO:0044425). Second, it was enriched in cellular process (GO:0009987), single-organism process (GO:0044699), biological regulation (GO:0065007), metabolic process (GO:0008152), and response to stimulus (GO:0050896) genes. There were fewer differentially expressed genes annotated in MF, which were concentrated in binding (GO:0005488), catalytic activity (GO:0003824), molecular transducer activity (GO:0060089), and receptor activity (GO:0004872). Additionally, the statistics of GO enrichment results show that the number of differential genes enriched in BP (1370 GO terms) is the largest, followed by MF (457 GO terms) and CC (278 GO terms). As shown in Supplementary Figure 1, the bubble diagram of BP enrichment shows that differentially expressed genes are primarily enriched in oxidation-reduction process (GO:0055114), RNA-dependent DNA biosynthetic process (GO:0006278), positive regulation of gene expression (GO:0010628), and regulation of the heart rate by cardiac conduction (GO:0086091).

**FIGURE 5:**
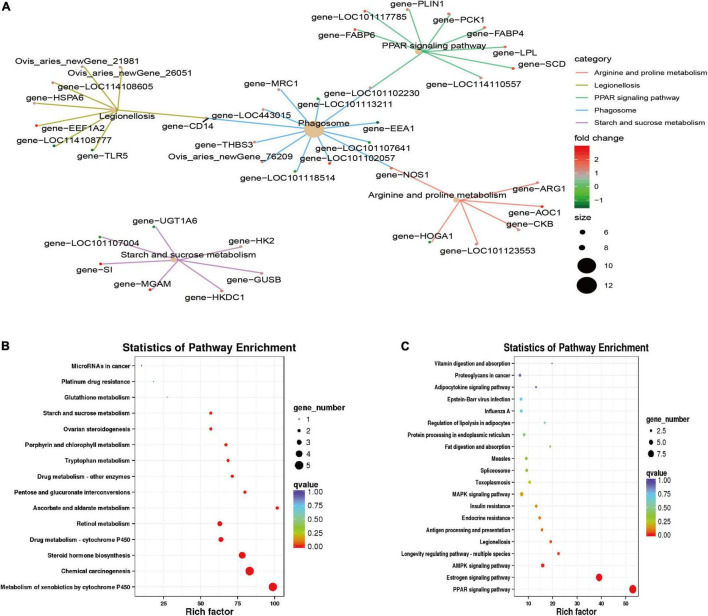
Kyoto Encyclopedia of Genes and Genomes (KEGG) feature enrichment. **(A)** KEGG enrichment map of all differential genes. **(B)** Up-regulation gene enrichment pathway analysis. **(C)** Down-regulation gene enrichment pathway analysis.

Kyoto Encyclopedia of Genes and Genomes enrichment analysis (*q* value < 0.05; Figure 5) found that the differentially expressed genes were primarily enriched in the PPAR signaling pathway (ko03320), legionellosis (ko05134), phagosome (ko04145), arginine and proline metabolism (ko00330), and the starch and sucrose metabolism (ko00500) pathways. Among them, the PPAR signaling pathway is a pathway related to lipid metabolism and gluconeogenesis; the phagosome and legionellosis pathways are primarily related to the body’s immune stress. Upregulated genes were significantly enriched in the metabolism of xenobiotics by cytochrome P450 (ko00980), chemical carcinogenesis (ko05204), steroid hormone biosynthesis (ko00140), retinol metabolism (ko00830) and other pathways; downregulated genes were primarily enriched in the PPAR signaling pathway (ko03320), estrogen signaling pathway (ko04915), AMPK signaling pathway (ko04152), and other pathways.

### Reverse Transcription-Quantitative PCR Verification

**FIGURE 6:**
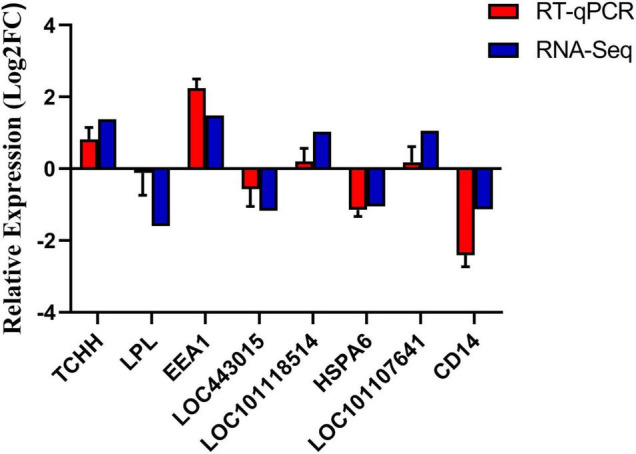
Verification of differentially expressed genes.

Eight genes were randomly selected for verification, of which four genes were up-regulated and four genes were down-regulated. As shown in Figure 6, the qPCR expression patterns of the selected genes are consistent with the RNA-Seq analysis results, indicating the reliability and accuracy of the RNA-Seq method used in this study.

### Analysis of Rumen Metabolome and Microbiome

#### Differential Metabolite Analysis

**FIGURE 7:**
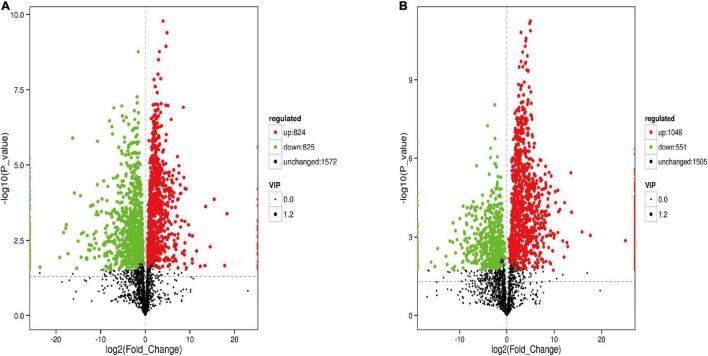
The volcano map of different metabolites in the rumen. **(A)** Positive ion model. **(B)** Negative ion model.

**FIGURE 8:**
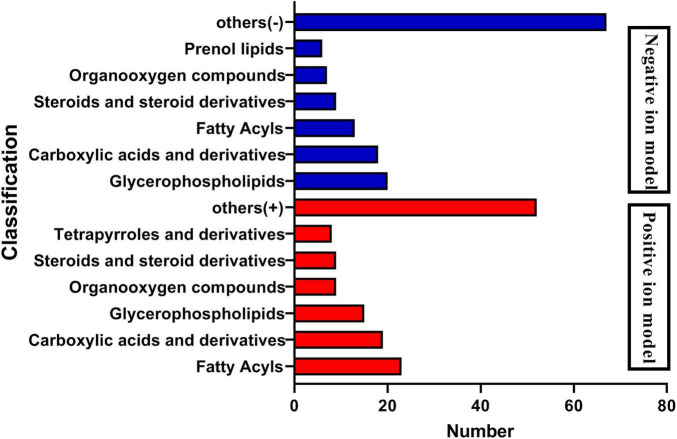
Classification diagram of differential metabolites.

The analysis of metabolites in the rumen during cold and warm seasons revealed 3,221 metabolites (positive model) and 3,104 metabolites (negative model). Principal component analysis (PCA) found that there were specific differences in the rumen metabolites of Tibetan sheep during cold and warm seasons under two different ion modes (positive and negative ions). Orthogonal partial least squares discriminant analysis (OPLS-DA) found that the R2X, R2Y, and Q2 values were close to 1, where Q2 > 0.9, further verifying the reliability of the OPLS-DA Model (Supplementary Figure 2). FC > 1, *P* value < 0.05, and VIP > 1 were used as the screening criteria to perform differential metabolite analysis (Figure 7). In the positive model, a total of 1,649 differential metabolites were identified, with 824 upregulated and 825 downregulated differential metabolites, of which 25 were unique differential metabolites during the cold season (Supplementary Material 2), such as hexanal, cynaroside A, and rishitin. In the negative model, a total of 1,599 differential metabolites were identified, with 1,048 upregulated and 551 downregulated differential metabolites, of which 131 were unique differential metabolites (sanguisorbin B, undecanedioic acid, geranyl acetone, tryptophyl-Valine, etc.). Further functional annotation of the differential metabolites showed that 145 (positive model) and 140 (negative model) differential metabolites were annotated to the KEGG function, and the annotations were classified and analyzed based on the HMDB database (Figure 8). We found 145 positive ionization differential metabolites, which were primarily classified into fatty acids (23), followed by carboxylic acids and derivatives (19), glycerophospholipids (15), etc. The 140 negatively ionized metabolites were primarily classified into glycerophospholipids (20), followed by carboxylic acids and derivatives (18) and fatty acyls (13).

#### Functional Annotation and Enrichment Analysis of Differential Metabolites

**FIGURE 9:**
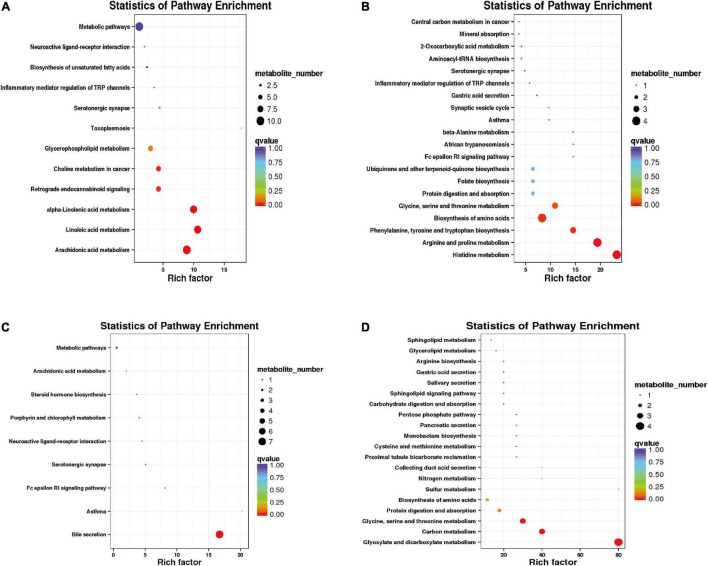
Functional enrichment analysis of differential metabolites. **(A)** Functional enrichment diagram of up-regulated differential metabolites under positive model. **(B)** Functional enrichment of up-regulated differential metabolites in a negative model. **(C)** Functional enrichment of down-regulated differential metabolites in a positive model. **(D)** Functional enrichment of down-regulated differential metabolites in a negative model.

In the positive mode, the differential metabolites between warm and cold seasons were primarily enriched in arachidonic acid metabolism, alpha-linolenic acid metabolism, linoleic acid metabolism, bile secretion and the metabolism of xenobiotics by cytochrome P450. In the negative mode, the differential metabolites were primarily enriched in purine metabolism, glycine, serine and threonine metabolism, folate biosynthesis, tryptophan metabolism and protein digestion and absorption (Supplementary Figure 3). The cold season-upregulated metabolic pathways enriched a total of 12 pathways in the positive mode (Figure 9A), among which arachidonic acid metabolism, linoleic acid metabolism, and alpha-linolenic acid metabolism were the primary enriched metabolic pathways, involving hexanal, lipoxin A4, alpha-linolenic acid, gamma-linolenic acid (*P* = 0.001904814), leukotriene A4, and other metabolites. A total of 23 metabolic pathways upregulated in the cold season were enriched in negative mode (Figure 9B), including histidine metabolism; arginine and proline metabolism; phenylalanine, tyrosine and tryptophan biosynthesis; biosynthesis of amino acids (*P* = 0.000643301); glycine, serine and threonine metabolism were significantly enriched metabolic pathways; and the differential metabolites primarily included histidine, arginine, proline, glycine, serine, and threonine. During the cold season, downregulated metabolic pathways were enriched in a total of 9 in the positive mode (Figure 9C). The significantly enriched metabolic pathway was the bile secretion (*P* < 0.001) pathway, and the primary differential metabolites were ibuprofen, lamivudine, leukotriene C4 and bilirubin. There were 27 metabolic pathways that were enriched in the negative mode (Figure 9D). The metabolic pathways that were significantly enriched were glyoxylate and dicarboxylate metabolism (*P* < 0.001), carbon metabolism (*P* = 0.00001), and glycine, serine and threonine metabolism (*P* = 0.00004). The different metabolites were primarily L-serine, glyceric acid, carbon dioxide, and 4-hydroxy-2-oxoglutaric acid.

#### Interaction Analysis of Rumen Metabolome and Microbiome

**FIGURE 10:**
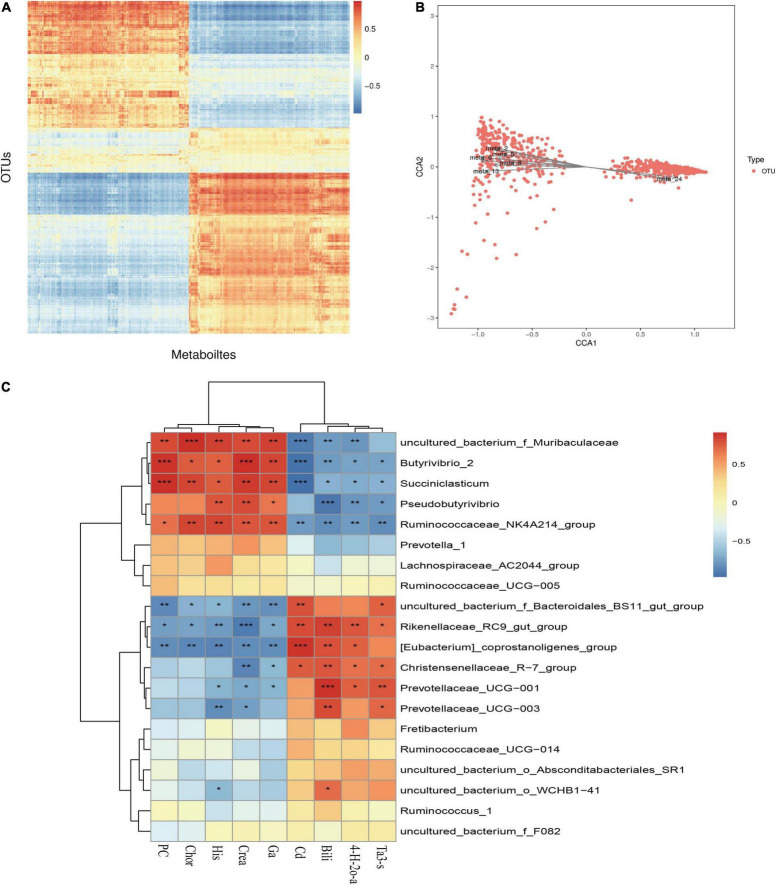
Correlation analysis of rumen microbes and metabolites. **(A)** Correlation analysis between differential metabolites and OUT. **(B)** Restriction correspondence analysis between differential metabolites and microflora. **(C)** Correlation heat map between differential metabolites and microflora. *P < 0.05; **P < 0.01; ***P < 0.001.

The results of previous studies on microbial diversity (Liu et al., 2020) and metabolome correlation analysis were combined to reveal the relationship between metabolites and microbial taxon OTUs to analyze the microbial population structure, physiological metabolism and genetic variation. As shown in Figure 10, the correlation heat map reveals that there is a specific correlation between different metabolites and microorganisms. This study screened microorganisms and metabolites with a correlation C value of greater than 0.99 and a CCP < 0.01 and found that *Christensenellaceae_R-7_group*, *Ruminiclostridium_9*, *Zoogloea*, and the metabolites avocadene 2-acetate, lysyl-asparagine, gamma-glutamyl cysteinylserine, and biocytin have extremely significant and strong correlations (*P* < 0.01). In addition, restricted correspondence analysis was performed for differential metabolites and microflora, as shown in the Figure 10B. Meta2 (lumochrome), meta5 (D-pyroglutamic acid), meta_6 (L-pipecolic acid), meta_8 [caprylic acid (octanoic acid)], Meta_13 (inosine), meta_24 (theasapogenol E), and other differential metabolites are closely related to microorganisms.

As shown in Figure 10C, a correlation analysis between the top 20 microorganisms at the genus level and the differential metabolites was further performed. Bilirubin was extremely significantly positively correlated with *Prevotellaceae_UCG-001* (*P* < 0.001) and extremely significantly negatively correlated with *Pseudobutyrivibrio* (*P* < 0.001). Creatine had a very significant negative correlation with *Rikenellaceae_RC9_gut_group* (*P* < 0.001) and a positive correlation with the difference in *Butyrivibrio_2* (*P* < 0.05). *Uncultured_bacterium_o_WCHB1-41* was positively correlated with bilirubin and negatively correlated with histamine. From the correlation heat map, it is clear that the dominant bacteria in the rumen of the cold season are positively correlated with the upregulated metabolites in the cold season, and the dominant bacteria in the rumen of the warm season are positively correlated with the downregulated metabolites. Further study found that the dominant bacteria *Rikenellaceae_RC9_gut_group* and carbon dioxide, bilirubin, 4-hydroxy-2-oxoglutaric acid, and taurolithocholic acid 3-sulfate were positively correlated but negatively correlated with creatine, guanidinoacetic acid, histamine, PC(20:5(5Z,8Z,11Z,14Z,17Z)/24:0) and chorismate.

### Rumen Transcriptome-Microbiome-Metabolome Joint Analysis

**FIGURE 11:**
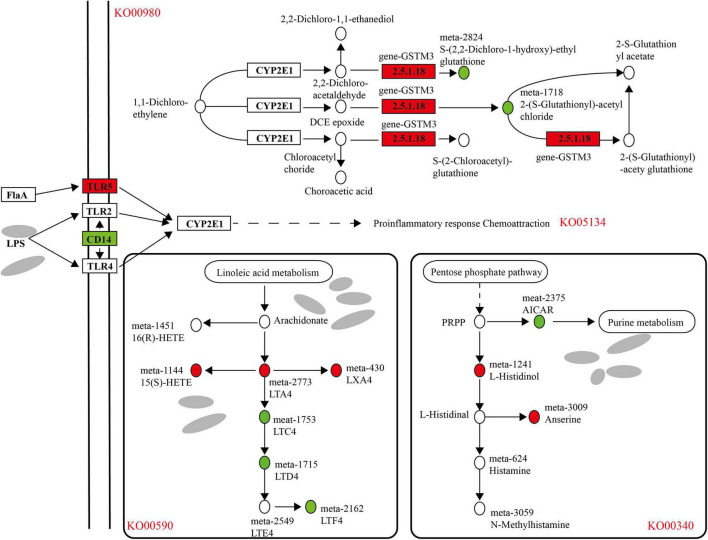
Analysis of differential genes and metabolite regulatory pathways in rumen epithelium during cold and warm seasons. Boxes represent genes, circles represent metabolites, red represents up-regulation and green represents down-regulation.

As shown in the figure, there was a correlation between the rumen microbial flora and the host transcriptome (Supplementary Figure 4); for example, the *Rikenellaceae-RC9-gut-group*, *Prevotallaceae-UCG-003*, *Butyrivibrio-2*, *Ruminococcaceae-NK4A214-group* and other flora had a significant correlation with host gene expression (*P* < 0.05). In the transcript of the rumen epithelium, the differential genes that were upregulated in the cold season were significantly enriched in metabolism of xenobiotics by cytochrome P450 (KO00980), and in metabolome studies, differential metabolites were also found to be enriched in this pathway, as shown in Figure 11, due to the metabolic process of cytochrome P450, the expression of gen-*GSTM3* is significantly upregulated in the cold season, and the metabolites S-(2,2-dichloro-1-hydroxy)-ethyl glutathione and 2-(*S*-glutathionyl)-acetyl chloride was significantly downregulated. In addition, the legionellosis (ko05134) pathway is involved in the immune stress response. Under the influence of the external environment and microbial metabolites, the expression of the *TLR5* gene is upregulated in the cold season, and the expression of the *CD14* gene is downregulated, which leads to the occurrence of proinflammatory response chemoattraction. In the microbial metabolome, the metabolites upregulated during the cold season were primarily enriched in the arachidonic acid metabolism and histidine metabolism pathways. The pathway diagram shows that linoleic acid metabolism produces arachidonate, which further produces LTA4, 15(S)-HETE, and LXA4, all of which are cold season upregulations. In the leukotriene metabolism pathway, it is metabolized into LTF4, which is downregulated during the cold season. In the histidine metabolism pathway, it participates in the pentose phosphate pathway, in which the cold-season downregulated metabolite AICAR nucleotide further participates in purine metabolism. L-Histidinol histamine alcohol was also upregulated and involved in the production of histamine metabolites as well as the production of anserine metabolites. In addition, we found that cold season-upregulated genes are also enriched in the steroid hormone biosynthesis (ko00140) and retinol metabolism (ko00830) pathways, resulting in the production of a large amount of glycosides and vitamin A and other metabolites (Supplementary Figures 5, 6).

## Discussion

In this study, we integrated rumen epithelial morphology, epithelial transcriptomics, microbiology and metabolomics analyses to explore the interaction between the rumen host and microbiota and their metabolites, thereby revealing the regulatory mechanism of Tibetan sheep adaptability to the plateau cold season. The rumen epithelium is composed of leaf-shaped papillae, which not only serve as an absorption structure but also serve as an epithelial barrier to prevent the invasion of rumen microorganisms or toxins (Graham and Simmons, 2005). Recent studies have indicated that the characteristics (size) of fiber particles and the effective fiber content play a key role in rumen muscularization and volume development (Rickard and Ternouth, 1965); the higher the NDF (Neutral detergent fiber) (such as hay) is, the better the development of the rumen muscle layer (Suárez et al., 2006). In this study, the muscular thickness of the rumen wall in the cold season was significantly higher than that in the warm season, which was consistent with previous research results and might be related to the fact that Tibetan sheep grazed a large amount of withered grass during the cold season. On the one hand, the rumen muscle layer is adapted to the stimulation of the dry withered grass, which leads to an increase in the thickness of the rumen muscle layer; on the other hand, ingesting a large amount of withered grass that is not easily degraded and digested, the body needs to increase the level of rumen movement to degrade and digest the withered grass, thereby promoting the development of the rumen muscle layer. In this study, under the stimulation of cold-season hay, the width of the rumen nipple in the cold season was significantly larger than that in the warm season, and the height of the papilla in the warm season was larger than that in the cold season, and because of the cold season hay was of poor quality, thus strengthened the movement of rumen nipple, resulting in cold season rumen nipple width and height change (Wang et al., 2017). The rumen epithelium is composed of the stratum corneum, granular layer, spinous layer and basal layer and has important physiological functions such as VFA (Volatile fatty acid) metabolism, absorption, transportation and protection (Gálfi et al., 1991; Steele et al., 2016). The stratum corneum has a small metabolic capacity and is an important protective barrier (da Silva et al., 2020), and this study found that the thickness of the stratum corneum during the cold season is significantly higher than that in the warm season. This finding may be due to the rough quality and high fiber content of the cold season. The rumen is strongly contracted and relaxed to complete mechanical digestion, which promotes an increase in the thickness of the stratum corneum, thereby forming a strong epithelial protective barrier. The basal layer is the most important rumen energy metabolism layer, and basal cells contribute to metabolic properties such as ketone production (Baldwin and Jesse, 1991). Granular layer cells establish tight gap junctions to maintain the integrity of the metabolite concentration gradient in the whole rumen wall (Baldwin and Connor, 2017). The results of this study indicated that the thickness of the granular layer during the cold season was significantly greater than that in the warm season, while the basal layer appeared to be more compact in the cold season. These special morphological structures provided the possibility for Tibetan sheep to adapt to the cold season. To understand the molecular mechanism behind this morphological difference, rumen epithelial tissue was further analyzed by RNA-seq.

Studies have found that *TCHH* and *TCHHL2* have homology, and *TCHHL2* plays a role in the cross-linking of keratin on the rumen surface (Segawa et al., 2014). In the abovementioned morphological studies, it was also found that the thickness of the stratum corneum increased during the cold season, which may be caused by the high expression of the *TCHH* gene. In addition, studies have found that *GSTM3* has the effects of detoxification and the removal of reactive oxygen species (ROS) (Wang et al., 2018, 2020), and under the harsh environment of the cold season, to ensure the normal function of the rumen. In this study, *DECR1* gene was highly expressed in the rumen in the cold season, and it was found that *DECR1* encodes the rate-limiting enzyme of polyunsaturated fatty acid (PUFAs) oxidation, which plays a certain role in disease treatment (Blomme et al., 2020). In conclusion, these genes with high expression in the cold season improve the immunity of Tibetan sheep, thus providing the possibility for Tibetan sheep to adapt to the cold season. In addition, we screened an important cold season-upregulated gene, *LOC101113965*. Studies have found that *LOC101113965* (ubiquitin) plays an important role in regulating protein function, and the dysregulation of the ubiquitin system leads to the occurrence of many diseases (Akutsu et al., 2016). The high expression in the rumen of the cold season regulates the protein in the rumen, thereby maintaining the normal function of the rumen. The further functional annotation analysis of its differential genes was performed, and in the GO database, most of the genes were enriched in processes related to cell growth and organelle growth, which was consistent with the results of Lin et al. (2019). In addition, we also found that differentially expressed genes were enriched in some processes related to biological regulation and metabolism and in response to some external environmental stimuli. We speculate that under the stimulation of the cold season environment, some biological processes and metabolic functions of the rumen epithelium will change to make corresponding physiological and behavioral responses to adapt to the special cold season environment. In addition, the KEGG functional enrichment analysis of differential genes indicated that they were primarily enriched in pathways related to lipid metabolism, gluconeogenesis, and body immunity. Studies have shown that rumen epithelial immune function is regulated differently during dietary transition (Zhao et al., 2017), which is consistent with the results of this study. Under the condition of nutrient deficiency in cold season, *CYP1A1*, *GSTM*, and other genes of cytochrome P450 (KO00980) pathway are highly expressed, leading to higher rumen toxin clearance ability of animals (Zhao et al., 2017). These genes are reportedly involved in the metabolism of specific rumen toxins (Nelson et al., 1996; Höög et al., 2003), indicating that under the influence of the harsh cold season environment, the rumen organs of Tibetan sheep have a greater ability to remove toxins, thereby maintaining normal physiological functions. Second, upregulated genes are enriched in steroid hormone biosynthesis (ko00140) and retinol metabolism (ko00830). Studies have shown that steroid hormones mediate various important developmental and physiological functions in different organs, and a lack of steroids can cause adverse effects (Schwartz et al., 2016). Adrenocortical hormone is a type of steroid hormone. It regulates glucose metabolism, inhibits the oxidation of sugar, raises blood sugar, and promotes the conversion of protein into sugar. Therefore, with the lack of nutrition during the cold season, the synthesis of steroid hormones ensures the body’s normal sugar metabolism balance. Vitamin A can increase incision inflammation and angiogenesis, repair collagen synthesis, and promote epithelial cell differentiation (Polcz and Barbul, 2019) to enhance intestinal resistance in the harsh environment of the cold season.

There is a strong symbiotic relationship between the animal intestinal microbiota and the host. The microbiota interacts with a variety of physiological functions in the host through its metabolites (Ren et al., 2018). Therefore, we further used GC-MS to analyze the metabolic functions of the rumen microbiota in the cold and warm seasons. In the positive/negative mode, 25 and 131 different metabolites unique to the cold season were found, among which cynaroside A, sanguisorbin B, tryptophyl-valine, and other metabolites were present in higher concentrations. Cynaroside is a flavonoid compound that is commonly found in honeysuckle (Hu et al., 2015). Recent studies have shown that cynaroside is absorbed in the gastrointestinal tract, has an antioxidant effect, inhibits the oxidation of lipids and proteins (Chen and Kang, 2014; Chang et al., 2016; de Lima et al., 2018; Grzegorczyk-Karolak et al., 2015), reduces cholesterol in atherosclerosis, and enhances capillary relaxation. In recent years, plant-derived bioactive compounds, such as saponins, have been considered potential alternatives to traditional antibiotics used as growth promoters in ruminant production (Benchaar et al., 2008). Saponins have the ability to regulate rumen microbial communities and rumen metabolites. Research has found that elms have anti-inflammatory effects (Chen et al., 2020). Studies have shown that valine is extensively metabolized in the rumen as a potential ketogenic and sugar-generating substance (Menahan and Schultz, 1964) and promotes growth (Gerulat and Berg, 1960). We speculate that these unique metabolites play an important role in cold season adaptation. Further functional annotation classification of these differential metabolites revealed that both positive and negative ion compounds were classified into fatty acids and some of their derivatives, indicating that the differential metabolites in the cold and warm seasons were primarily fatty acid-related metabolites that participate in the body energy process. Among them, the positive ion metabolites that were upregulated during the cold season were primarily enriched in arachidonic acid metabolism, linoleic acid metabolism, alpha-linolenic acid metabolism and other pathways. Caldari-Torres et al. (2011) found that the lipids of natural ruminant forages contain a large amount of essential linolenic acid, and linoleic acid can reduce cardiovascular disease (Dewhurst et al., 2006). Arachidonic acid is a biologically active substance of many cyclic eicosanic acid derivatives, and it is a ubiquitous component in mammalian cells. It is primarily found in glycerolipids or glycerophospholipids (Dedyukhina et al., 2015; Martin et al., 2016), and it is not only a tetra-unsaturated fatty acid that is important for normal cell membrane fluidity but also plays other important biochemical roles, including as direct precursors of bioactive lipid mediators, such as prostaglandins and leukotrienes (Liu et al., 2019). These metabolic pathways were significantly upregulated in the cold season, suggesting that Tibetan sheep used essential fatty acids such as arachidonic acid, linoleic acid and linolenic acid to catabolize and adapt to nutritional stress and harsh environmental conditions in the cold season and to cope with nutritional stress and a low-temperature environment in the cold season. The anionic metabolites that were upregulated during the cold season were primarily enriched in some amino acid-related metabolic pathways. The amino acids in the rumen are the key precursors for protein and peptide synthesis and are primarily derived from dietary protein and trace protein (Mariz et al., 2018), and rumen microbial proteins produced by rumen microorganisms can meet 90% of the amino acids that reach the small intestine (Flint and Bayer, 2008). Among them, arginine and proline are involved in RNA synthesis and protein glycosylation, which are necessary for cell function (Elolimy et al., 2020). Proline is the main amino acid that maintains cell structure and function, and it is also an important regulator of cell metabolism and physiology. In addition, proline plays an important role in the synthesis and structure of proteins, metabolism and nutrition as well as the antioxidant response in trauma and immune responses (Wu et al., 2011). In addition, the cationic metabolites that were downregulated during the cold season were primarily enriched in the bile secretion pathway. The differential metabolite bilirubin is the product of bile metabolism. Bile protects the body from intestinal infections by secreting immunoglobulin A (IgA) and inflammatory cytokines and stimulating the innate immune system of the intestines (Boyer, 2013). In the harsh environment of the cold season, the secretion of bile enhances the immune function of the rumen epithelium. According to research findings, the gut microbiota interacts with a variety of physiological functions in the host body through its metabolites (Ren et al., 2018). Therefore, this study combined the results of previous microbial studies, provided a correlation analysis with the metabolome, and found that there is a correlation between different metabolites and microbes. The correlation analysis shows that *Rikenellaceae_RC9_gut_group* and *Christensenellaceae_R-7* are associated with creatine and guanidinoacetic acid, respectively. It has a significant negative correlation with histamine and other metabolites. Studies have found that an increase in the abundance of the genus *Christensenellaceae_R-7* enhances the catabolism of arginine (Wang B. et al., 2019). Citrulline, a precursor for the synthesis of arginine, is positively related to *Christensenellaceae_R-7* (Wang B. et al., 2019). Arginine can synthesize guanidinoacetic acid and creatine (Takeda et al., 1992). This result further illustrates that the abundance of *Christensenellaceae_R-7* is negatively correlated with the content of guanidinoacetic acid and creatine (*p* < 0.05). The addition of saponins to the diet resulted in a decrease in the abundance of *Prevotella_1* and *Christensenellaceae_R-7*, while alfalfa contained a large amount of saponins (Sen et al., 1998; Wang B. et al., 2019), further verifying that the abundance of *Christensenellaceae_R-7* during the cold season increased significantly.

Based on the above analysis, we found that there is a correlation between the rumen epithelial transcriptome, microbiome and metabolome. The correlation analysis found that in the metabolism of xenobiotics by the cytochrome P450 (KO00980) pathway, the expression of gen*-GSTM3* was significantly upregulated in the cold season, resulting in the metabolites *S*-(2,2-dichloro-1-hydroxy)-ethyl glutathione and 2-(*S*-glutathionyl)-acetyl chloride being significantly downregulated. The cytochrome P450 metabolic pathway plays a key role in the detoxification, cell metabolism and homeostasis of exogenous drugs (Manikandan and Nagini, 2018). Studies have found that *GSTM3* belongs to the exogenous detoxification phase II enzyme family, and the detoxification of carcinogens is related to the metabolism of exogenous electrophilic substances. In addition, the legionellosis (ko05134) pathway is involved in the response to immune stress. Under the influence of the external environment and microbial metabolites, the expression of the *TLR5* gene is upregulated in the cold season, and the expression of the *CD14* gene is downregulated, which leads to the occurrence of proinflammatory response chemoattraction. *TLR5* binds to bacterial flagellin to trigger the innate immune response to invading pathogens (Yoon et al., 2012). The human monocyte differentiation antigen *CD14* is a pattern recognition receptor (PRR) that can enhance the innate immune response, and it has been confirmed that *CD14* is a *TLR* co-receptor for detecting pathogen-related molecular patterns (Wu et al., 2019). The enrichment of the legionellosis (ko05134) pathway in this study indicates that the innate immune mechanism of Tibetan sheep during the cold season has been improved. The pathway diagram shows that linoleic acid metabolism produces arachidonate arachidonic acid, which further produces LTA4, 15(S)-HETE, and LXA4, all of which are upregulated during the cold season. Lipoxin A4 (LXA4), one of the earliest endogenous lipid mediators, can inhibit the accumulation of neutrophils and inhibit the occurrence of inflammation (Zhu et al., 2020). In the histidine metabolism pathway, it participates in the pentose phosphate pathway, in which the cold-season downregulated metabolite AICAR nucleotide further participates in purine metabolism. L-Histidinol was also upregulated and involved in the production of histamine metabolites. Many tissues, especially the mast cells of the skin, lung and intestinal mucosa, contain large amounts of histamine. When tissues are damaged or inflammation and allergic reactions occur, histamine can be released, and vasodilation and vascular permeability can be generated through the H1 and H2 receptors (Greaves and Sabroe, 1996). These enrichment pathways could provide for the adaptability of Tibetan sheep to the cold season. In addition, we found that the cold season-upregulated genes were also enriched in the steroid hormone biosynthesis (ko00140) and retinol metabolism (ko00830) pathways, producing a large amount of glycosides and vitamin A and other metabolites, providing cold season Tibetan sheep with some energy requirements.

## Conclusion

Under the stimulus of the high-altitude cold season environment, the transcription status of the rumen epithelium and the rumen microbes and their metabolites of Tibetan sheep have undergone adaptive changes, resulting in changes in the morphological structure of the rumen epithelium. A transcriptomics analysis of the rumen epithelium indicated that differentially expressed genes were enriched in pathways related to cell and organelle growth in the rumen epithelium, which may lead to changes in rumen epithelial morphology. KEGG functional enrichment analysis indicated that differentially expressed genes were primarily enriched in pathways related to lipid metabolism, gluconeogenesis, and body immunity, thereby improving the immune function of Tibetan sheep during the cold season. In addition, cold-season differential microbes interact with a variety of physiological functions in the host through their metabolites. During the cold season, cynaroside A, sanguisorbin B, and tryptophyl-valine unique metabolites play a vital role in the antioxidation, anti-inflammatory, and growth processes of the rumen epithelium. Functional annotation analysis indicated that these differential metabolites play important roles in cell metabolism, physiological regulation and immunity. A comprehensive transcriptome-metabonomics-microbiome pathway analysis showed that during the metabolism of xenobiotics by the cytochrome P450 (KO00980) pathway, the expression of the epithelial gen GSTM3 was upregulated in the cold season, leading to the downregulation of some harmful metabolites. Therefore, the rumen organs of Tibetan sheep showed a higher ability to remove toxins physiologically. Under stimulation by the external environment and microbial metabolites, the expression of the *TLR5* gene in the legionellosis (ko05134) pathway was upregulated in the cold season, and the expression of the *CD14* gene was downregulated, which led to the improvement of the innate immune function of Tibetan sheep during the cold season. In addition to the steroid hormone biosynthesis (ko00140) and retinol metabolism (ko00830) pathways, a large amount of glycosides and vitamin A and other metabolites are produced, which provide for specific energy requirements for cold season Tibetan sheep. These key pathways provide insight into the molecular and metabolic mechanisms behind the cold season adaptability of Tibetan sheep.

## Data Availability Statement

The datasets presented in this study can be found in online repositories. The names of the repository/repositories and accession number(s) can be found below: Sequence Read Archive (SRA): SRR17883805–SRR17883810/SRR12719079–SRR12719088.

## Ethics Statement

The animal study was reviewed and approved by Livestock Care Committee of Gansu Agricultural University.

## Author Contributions

YS, XL, YL, and JH designed the study. YS, WL, XG, XP, JW, and SL performed the experiments and collected the samples. YS and XL analyzed the data and wrote the manuscript. All authors contributed to manuscript revision, read and approved the final version.

## Conflict of Interest

The authors declare that the research was conducted in the absence of any commercial or financial relationships that could be construed as a potential conflict of interest.

## Publisher’s Note

All claims expressed in this article are solely those of the authors and do not necessarily represent those of their affiliated organizations, or those of the publisher, the editors and the reviewers. Any product that may be evaluated in this article, or claim that may be made by its manufacturer, is not guaranteed or endorsed by the publisher.

## Footnotes

1
www.biocloud.net

## Supplementary Material

Supplementary Material 1 Analysis of differential genes in rumen transcriptome in cold and warm seasons.

Supplementary Material 2 Analysis of differential metabolites in cold and warm seasons.

## References

[B1] Akutsu M. Dikic I. Bremm A. (2016). Ubiquitin chain diversity at a glance. *J. Cell Sci.* 129 875–880. 10.1242/jcs.183954 26906419

[B2] Allan C. Stankey G. (2009). *Adaptive Environmental Management: a Practitioner’s Guide.* Netherlands: Springer.

[B3] Baldwin T. R. L. Connor E. E. (2017). Rumen Function and Development. The Veterinary clinics of North America. *Food Anim. Pract.* 33 427–439. 10.1016/j.cvfa.2017.06.001 28807474

[B4] Baldwin T. R. L. Jesse B. W. (1991). Technical note: isolation and characterization of sheep ruminal epithelial cells. *J. Cell Sci.* 69 3603–3609. 10.2527/1991.6993603x 1938645

[B5] Benchaar C. Calsamiglia S. Chaves A. V. Fraser G. R. Colombatto D. McAllister T. A. (2008). A review of plant-derived essential oils in ruminant nutrition and production. *Anim. Feed Sci. Technol.* 145 209–228. 10.1016/j.anifeedsci.2007.04.014

[B6] Blomme A. Ford C. A. Mui E. Patel R. Ntala C. Jamieson L. E. (2020). 2, 4-dienoyl-CoA reductase regulates lipid homeostasis in treatment-resistant prostate cancer. *Nat. Commun.* 11:2508. 10.1038/s41467-020-16126-7 32427840 PMC7237503

[B7] Boyer J. L. (2013). Bile formation and secretion. *Compr. Physiol.* 3 1035–1078. 10.1002/cphy.c120027 23897680 PMC4091928

[B8] Caldari-Torres C. Lock A. L. Staples C. R. Badinga L. (2011). Performance, metabolic, and endocrine responses of periparturient Holstein cows fed 3 sources of fat. *J. Dairy Sci.* 94 1500–1510. 10.3168/jds.2010-3748 21338814

[B9] Chang J. Yao X. Zou H. Wang L. Lu Y. Zhang Q. (2016). BDNF/PI3K/Akt and Nogo-A/RhoA/ROCK signaling pathways contribute to neurorestorative effect of Houshiheisan against cerebral ischemia injury in rats. *J. Ethnopharmacol.* 194 1032–1042. 10.1016/j.jep.2016.11.005 27833029

[B10] Chen J. F. Tan L. Ju F. Kuang Q. X. Yang T. L. Deng F. (2020). Phenolic glycosides from Sanguisorba officinalis and their anti-inflammatory effects. *Nat. Prod. Res.* 18 1–8. 10.1080/14786419.2020.1849202 33205667

[B11] Chen L. Kang Y. -H. (2014). Antioxidant and Enzyme Inhibitory Activities of Plebeian Herba (Salvia plebeia R. Br.) under Different Cultivation Conditions. *J. Agric. Food Chem.* 62 2190–2197. 10.1021/jf404570s 24422962

[B12] da Silva T. G. P. Batista ÂM. V. Guim A. Da Silva J. V. A. de Carvalho F. F. R. de barros M. E. G. (2020). Histomorphometric changes of the fore-stomach of lambs fed with diets containing spineless cactus genotypes resistant to Dactylopius sp. *Trop. Anim. Health Prod.* 52 1299–1307. 10.1007/s11250-019-02129-0 31848832

[B13] de Lima R. Guex C. G. Da Silva A. R. H. Lhamas C. L. Dos Santos Moreira K. L. Casoti R. (2018). Acute and subacute toxicity and chemical constituents of the hydroethanolic extract of Verbena litoralis Kunth. *J. Ethnopharmacol.* 224 76–84. 10.1016/j.jep.2018.05.012 29772354

[B14] Dedyukhina E. G. Chistyakova T. I. Mironov A. A. Kamzolova S. V. Minkevich I. G. Vainshtein M. B. (2015). [The effect of pH, aeration, and temperature on arachidonic acid synthesis by Mortierella alpina]. *Prikl. Biokhim. Mikrobiol.* 51 243–250. 10.7868/s055510991502004x 26027361

[B15] Dewhurst R. J. Shingfield K. J. Lee M. Scollan N. D. (2006). Increasing the concentrations of beneficial polyunsaturated fatty acids in milk produced by dairy cows in high-forage systems. *Anim. Feed Sci. Technol.* 131 168–206. 10.1016/j.anifeedsci.2006.04.016

[B16] Elolimy A. Alharthi A. Zeineldin M. Parys C. Loor J. J. (2020). Residual feed intake divergence during the preweaning period is associated with unique hindgut microbiome and metabolome profiles in neonatal Holstein heifer calves. *J. Anim. Sci. Biotechnol.* 11:13. 10.1186/s40104-019-0406-x 31988748 PMC6972010

[B17] Flint H. J. Bayer E. A. (2008). Plant Cell Wall Breakdown by Anaerobic Microorganisms from the Mammalian Digestive Tract. *Ann. N. Y. Acad. Sci.* 1125 280–288. 10.1196/annals.1419.022 18378598

[B18] Gálfi P. Neogrády S. Sakata T. (1991). Effects of Volatile Fatty Acids on the Epithelial Cell Proliferation of the Digestive Tract and Its Hormonal Mediation. San Diego: Academic Press, Inc.

[B19] Gerulat B. F. Berg C. P. (1960). Growth promotion by D-valine and D-leucine. *Arch. Biochem. Biophys.* 88 273–279. 10.1016/0003-9861(60)90235-6 13827643

[B20] Graham C. Simmons N. L. (2005). Functional organization of the bovine rumen epithelium. *Am. J. Physiol. Regulat. Integr. Compar. Physiol.* 288 R173–R181. 10.1152/ajpregu.00425.2004 15319221

[B21] Greaves M. W. Sabroe R. A. (1996). Histamine: the Quintessential Mediator. *JAMA Dermatol.* 23 735–740. 10.1111/j.1346-8138.1996.tb02694.x 8990694

[B22] Grzegorczyk-Karolak I. Wysokińska H. Olas B. (2015). Studies on the antioxidant properties of extracts from the roots and shoots of two Scutellaria species in human blood plasma. *Acta Biochim. Polon.* 62 253–258. 10.18388/abp.2014_944 26015995

[B23] Hess M. Sczyrba A. Egan R. Kim T. W. Chokhawala H. Schroth G. (2011). Metagenomic Discovery of Biomass-Degrading Genes and Genomes from Cow Rumen. *Science* 331 463–467. 10.1126/science.1200387 21273488

[B24] Höög J. Strömberg P. Hedberg J. J. Griffiths W. J. (2003). The mammalian alcohol dehydrogenases interact in several metabolic pathways. *Chem. Biol. Interact.* 143 175–181. 10.1016/s0009-2797(02)00225-9 12604202

[B25] Hu R. Zou H. Wang Z. Cao B. Peng Q. Jing X. (2019). Nutritional Interventions Improved Rumen Functions and Promoted Compensatory Growth of Growth-Retarded Yaks as Revealed by Integrated Transcripts and Microbiome Analyses. *Front. Microbiol.* 10:318. 10.3389/fmicb.2019.00318 30846981 PMC6393393

[B26] Hu X. Huang W. Yang Y. (2015). Cytochrome P450 isoenzymes in rat and human liver microsomes associate with the metabolism of total coumarins in Fructus Cnidii. *Eur. J. Drug Metab Pharmacokinet.* 40 373–377. 10.1007/s13318-014-0219-4 24993184

[B27] Kim D. Langmead B. Salzberg S. L. (2015). HISAT: a fast spliced aligner with low memory requirements. *Nat. Methods* 12 357–360. 10.1038/nmeth.3317 25751142 PMC4655817

[B28] Kurilshikov A. Wijmenga C. Fu J. Zhernakova A. (2017). Host Genetics and Gut Microbiome: challenges and Perspectives. *Trends Immunol.* 38 633–647. 10.1016/j.it.2017.06.003 28669638

[B29] Li J. Ma W. Zeng P. Wang J. Geng B. Yang J. (2015). LncTar: a tool for predicting the RNA targets of long noncoding RNAs. *Brief Bioinform.* 16 806–812. 10.1093/bib/bbu048 25524864

[B30] Lin L. Xie F. Sun D. Liu J. Zhu W. Mao S. (2019). Ruminal microbiome-host crosstalk stimulates the development of the ruminal epithelium in a lamb model. *Microbiome* 7:83. 10.1186/s40168-019-0701-y 31159860 PMC6547527

[B31] Liu C. Wu H. Liu S. Chai S. Meng Q. Zhou Z. (2019). Dynamic Alterations in Yak Rumen Bacteria Community and Metabolome Characteristics in Response to Feed Type. *Front. Microbiol.* 10:1116. 10.3389/fmicb.2019.01116 31191470 PMC6538947

[B32] Liu X. Sha Y. Dingkao R. Zhang W. Lv W. Wei H. (2020). Interactions Between Rumen Microbes, VFAs, and Host Genes Regulate Nutrient Absorption and Epithelial Barrier Function During Cold Season Nutritional Stress in Tibetan Sheep. *Front. Microbiol.* 11:593062. 10.3389/fmicb.2020.593062 33250882 PMC7674685

[B33] Love M. I. Huber W. Anders S. (2014). Moderated estimation of fold change and dispersion for RNA-seq data with DESeq2. *Genome Biol.* 15:550. 10.1186/s13059-014-0550-8 25516281 PMC4302049

[B34] Malmuthuge N. Liang G. Guan L. L. (2019). Regulation of rumen development in neonatal ruminants through microbial metagenomes and host transcriptomes. *Genome Biol.* 20:172. 10.1186/s13059-019-1786-0 31443695 PMC6708143

[B35] Manikandan P. Nagini S. (2018). Cytochrome P450 Structure, Function and Clinical Significance: a Review. *Curr. Drug Targets* 19 38–54. 10.2174/1389450118666170125144557 28124606

[B36] Mariz L. D. S. Amaral P. M. Valadares Filho S. C. Santos S. A. Detmann E. Marcondes M. I. (2018). Dietary protein reduction on microbial protein, amino acid digestibility, and body retention in beef cattle: 2. Amino acid intestinal absorption and their efficiency for whole-body deposition. *J. Anim. Sci.* 96 670–683. 10.1093/jas/sky018 29385609 PMC6140959

[B37] Martin S. A. Brash A. R. Murphy R. C. (2016). The discovery and early structural studies of arachidonic acid. *J. Lipid Res.* 57 1126–1132. 10.1194/jlr.R068072 27142391 PMC4918860

[B38] Menahan L. A. Schultz L. H. (1964). Metabolism of Leucine and Valine within the Rumen1,2. *J. Dairy Sci.* 47 1080–1085. 10.3168/jds.S0022-0302(64)88849-4

[B39] Muegge B. D. Kuczynski J. Knights D. Clemente J. C. González A. Fontana L. (2011). Diet drives convergence in gut microbiome functions across mammalian phylogeny and within humans. *Science* 332 970–974. 10.1126/science.1198719 21596990 PMC3303602

[B40] Nelson D. R. Koymans L. Kamataki T. Stegeman J. J. Feyereisen R. Waxman D. J. (1996). P450 superfamily: update on new sequences, gene mapping, accession numbers and nomenclature. *Pharmacogenetics* 6 1–42. 10.1097/00008571-199602000-00002 8845856

[B41] Polcz M. E. Barbul A. (2019). The Role of Vitamin A in Wound Healing. *Nutr. Clin Pract.* 34 695–700. 10.1002/ncp.10376 31389093

[B42] Ren W. Wang P. Yan J. Liu G. Zeng B. Hussain T. (2018). Melatonin alleviates weanling stress in mice: involvement of intestinal microbiota. *J. Pineal. Res.* 64:e12448. 10.1111/jpi.12448 28875556

[B43] Rickard M. D. Ternouth J. H. (1965). The effect of the increased dietary volatile fatty acids on the morphological and physiological development of lambs with particular reference to the rumen. *J. Agric. Sci.* 65 371–377. 10.1017/S0021859600048930

[B44] Schwartz N. Verma A. Bivens C. B. Schwartz Z. Boyan B. D. (2016). Rapid steroid hormone actions via membrane receptors. *Biochim. Biophys. Acta* 1863 2289–2298. 10.1016/j.bbamcr.2016.06.004 27288742

[B45] Segawa K. Kurata S. Yanagihashi Y. Brummelkamp T. R. Matsuda F. Nagata S. (2014). Caspase-mediated cleavage of phospholipid flippase for apoptotic phosphatidylserine exposure. *Science* 344 1164–1168. 10.1126/science.1252809 24904167

[B46] Sen S. Makkar H. P. S. Becker K. (1998). Alfalfa saponins and their implication in animal nutrition. *J. Agric. Food Chem.* 46 131–140. 10.1021/jf970389i 10554208

[B47] Steele M. A. Penner G. B. Chaucheyras-Durand F. Guan L. L. (2016). Development and physiology of the rumen and the lower gut: targets for improving gut health. *J. Dairy Sci.* 99 4955–4966. 10.3168/jds.2015-10351 26971143

[B48] Suárez B. J. Van Reenen C. G. Gerrits W. J. Stockhofe N. van Vuuren A. M. Dijkstra J. (2006). Effects of supplementing concentrates differing in carbohydrate composition in veal calf diets: II. Rumen development. *J. Dairy Sci.* 89 4376–4386. 10.3168/jds.S0022-0302(06)72484-5 17033025

[B49] Takeda M. Kiyatake I. Koide H. Jung K. Y. Endou H. (1992). Biosynthesis of guanidinoacetic acid in isolated renal tubules. *Eur. J. Clin. Chem. Clin. Biochem.* 30:325. 10.1515/cclm.1992.30.6.325 1511066

[B50] Trapnell C. Mortazavi A. Kwan G. Williams B. A. Salzberg S. L. Pertea G. (2010). Transcript assembly and quantification by RNA-Seq reveals unannotated transcripts and isoform switching during cell differentiation. *Nat. Biotechnol.* 28 511–515. 10.1038/nbt.1621 20436464 PMC3146043

[B51] Wang B. Ma M. P. Diao Q. Y. Tu Y. (2019). Saponin-Induced Shifts in the Rumen Microbiome and Metabolome of Young Cattle. *Front. Microbiol.* 10:356. 10.3389/fmicb.2019.00356 30873143 PMC6403146

[B52] Wang B. Wang D. Wu X. Cai J. Liu M. Huang X. (2017). Effects of dietary physical or nutritional factors on morphology of rumen papillae and transcriptome changes in lactating dairy cows based on three different forage-based diets. *BMC Genom.* 18:353. 10.1186/s12864-017-3726-2 28477620 PMC5420399

[B53] Wang Q. Wang K. Wu W. Giannoulatou E. Ho J. W. K. Li L. (2019). Host and microbiome multi-omics integration: applications and methodologies. *Biophys. Rev.* 11 55–65. 10.1007/s12551-018-0491-7 30627872 PMC6381360

[B54] Wang S. Yang J. You L. Dai M. Zhao Y. (2020). GSTM3 Function and Polymorphism in Cancer: emerging but Promising. *Cancer Manag. Res.* 12 10377–10388. 10.2147/CMAR.S272467 33116892 PMC7585806

[B55] Wang Y. Yang Z. Y. Chen Y. H. Li F. Shen H. Yu Y. (2018). A novel functional polymorphism of GSTM 3 reduces clear cell renal cell carcinoma risk through enhancing its expression by interfering miR-556 binding. *J. Cell. Mol. Med.* 22 3005–3015. 10.1111/jcmm.13528 29569387 PMC5980204

[B56] Wu G. Bazer F. W. Burghardt R. C. Johnson G. A. Kim S. W. Knabe D. A. (2011). Proline and hydroxyproline metabolism: implications for animal and human nutrition. *Amino Acids* 40 1053–1063. 10.1007/s00726-010-0715-z 20697752 PMC3773366

[B57] Wu Z. Zhang Z. Lei Z. Lei P. (2019). CD14: biology and role in the pathogenesis of disease. *Cytokine Growth Fact. Rev.* 48 24–31. 10.1016/j.cytogfr.2019.06.003 31296363

[B58] Xue M. Sun H. Wu X. Liu J. Guan L. L. (2020). Multi-omics reveals that the rumen microbiome and its metabolome together with the host metabolome contribute to individualized dairy cow performance. *Microbiome* 8:64. 10.1186/s40168-020-00819-8 32398126 PMC7218573

[B59] Yoon S. I. Kurnasov O. Natarajan V. Hong M. Gudkov A. V. Osterman A. L. (2012). Structural basis of TLR5-flagellin recognition and signaling. *Science* 335 859–864. 10.1126/science.1215584 22344444 PMC3406927

[B60] Young M. D. Wakefield M. J. Smyth G. K. Oshlack A. (2010). Gene ontology analysis for RNA-seq: accounting for selection bias. *Genome Biol.* 11:R14. 10.1186/gb-2010-11-2-r14 20132535 PMC2872874

[B61] Zhang Z. Xu D. Wang L. Hao J. Wang J. Zhou X. (2016). Convergent Evolution of Rumen Microbiomes in High-Altitude Mammals. *Curr. Biol.* 26 1873–1879. 10.1016/j.cub.2016.05.012 27321997

[B62] Zhao K. Chen Y. H. Penner G. B. Oba M. Guan L. L. (2017). Transcriptome analysis of ruminal epithelia revealed potential regulatory mechanisms involved in host adaptation to gradual high fermentable dietary transition in beef cattle. *BMC Genom.* 18:976. 10.1186/s12864-017-4317-y 29258446 PMC5735905

[B63] Zhernakova A. Kurilshikov A. Bonder M. J. Tigchelaar E. F. Schirmer M. Vatanen T. (2016). Population-based metagenomics analysis reveals markers for gut microbiome composition and diversity. *Science* 352 565–569. 10.1126/science.aad3369 27126040 PMC5240844

[B64] Zhu J. J. Yu B. Y. Fu C. C. He M. Z. Zhu J. H. Chen B. W. (2020). LXA4 protects against hypoxic-ischemic damage in neonatal rats by reducing the inflammatory response via the IkappaB/NF-kappaB pathway. *Int. Immunopharmacol.* 89:107095. 10.1016/j.intimp.2020.107095 33096360

