# Supplementary material for: Multi-Omics Reveals That the Rumen Transcriptome, Microbiome, and Its Metabolome Co-regulate Cold Season Adaptability of Tibetan Sheep
Source: Front Microbiol. 2022 Apr 13;13:859601. doi: 10.3389/fmicb.2022.859601 (PMC9043902; doi:10.3389/fmicb.2022.859601)
Supplement: Supplementary file 1 [file Data_Sheet_1.docx]

Table S1 Primer information

| Gene | Primer（5'-3'） |
| --- | --- |
| TCHH | F:TTGGGCCGATTACCTGTGAG |
|  | R:ACCTCTTTCCTGCATTGCCT |
| LPL | F:GCGATTCCTACTTCAGCTGGT |
|  | R:TCCCGGGAACAGAAGATAACC |
| EEA1 | F:TGACCTTGGAACGTGAGAGAG |
|  | R:GTTCACTCCTCAGCTGGCTT |
| LOC443015 | F:TACGGAGACCTGAACCACCT |
|  | R:ATGAAGAAGTGGAGGCGTGG |
| LOC101118514 | F:AGATGCTGAGTGGTGTTGGG |
|  | R:CGCACAAGCCCCTTCTGG |
| HSPA6 | F:TAAATGGCCCCTCTGAACGG |
|  | R:CCACCCCTGAAGTTAGGCAG |
| LOC101107641 | F:GGATCTGAACACGCGGAGAA |
|  | R:CGCTCTGGTTGTAGTAGCCG |
| CD14 | F:CCCTCAGTCTCCGTAACGTG |
|  | R:GTGTGCTTGGGCAATGTTCA |


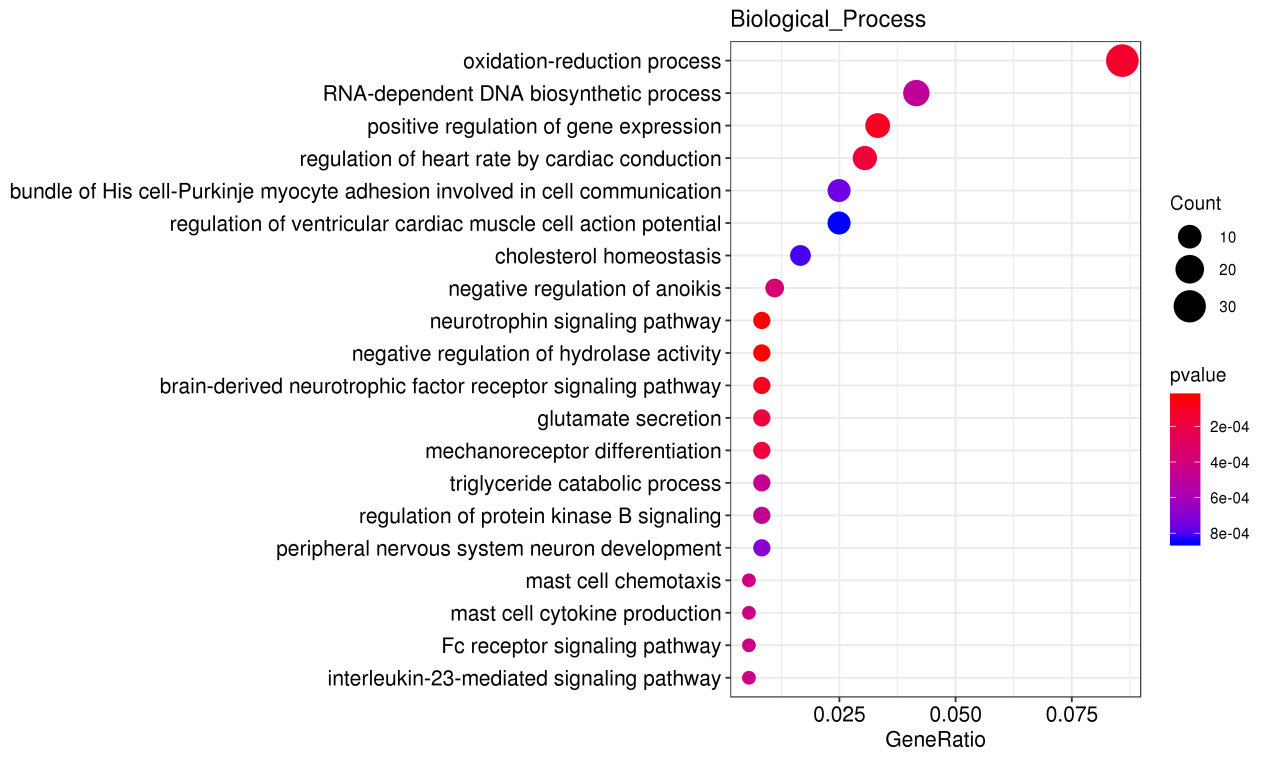


**Fig. S1** BP enrichment bubble chart


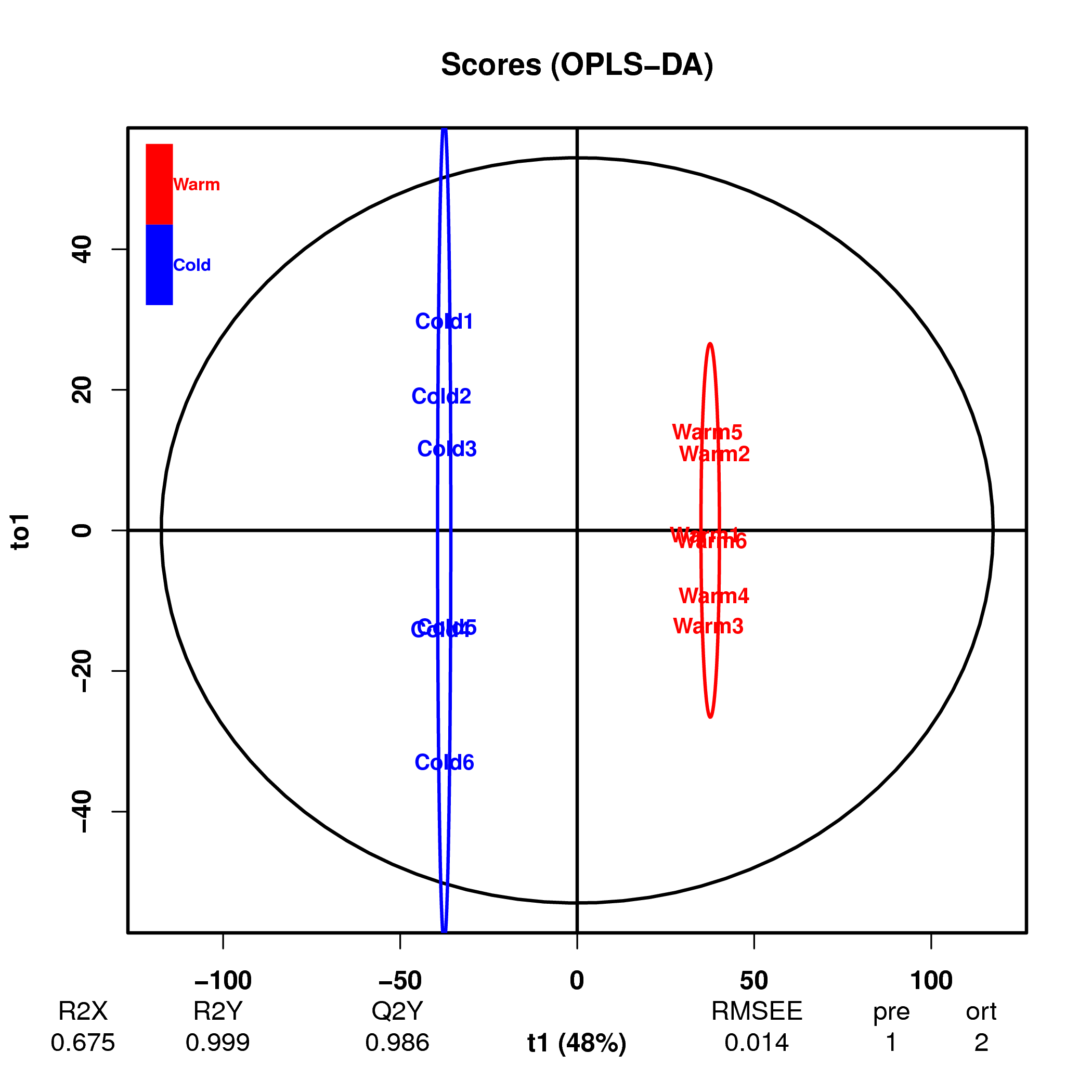

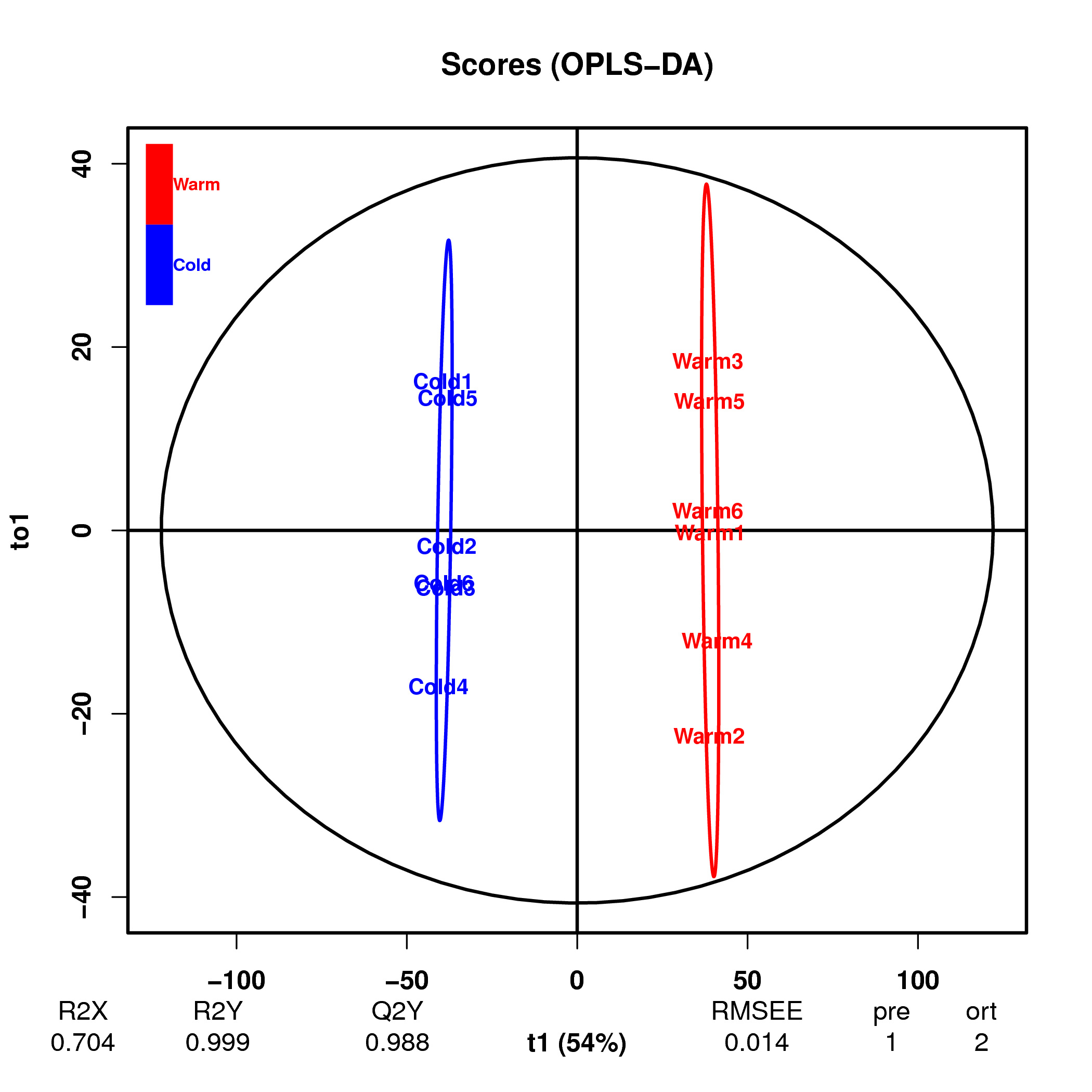


**Fig S2**


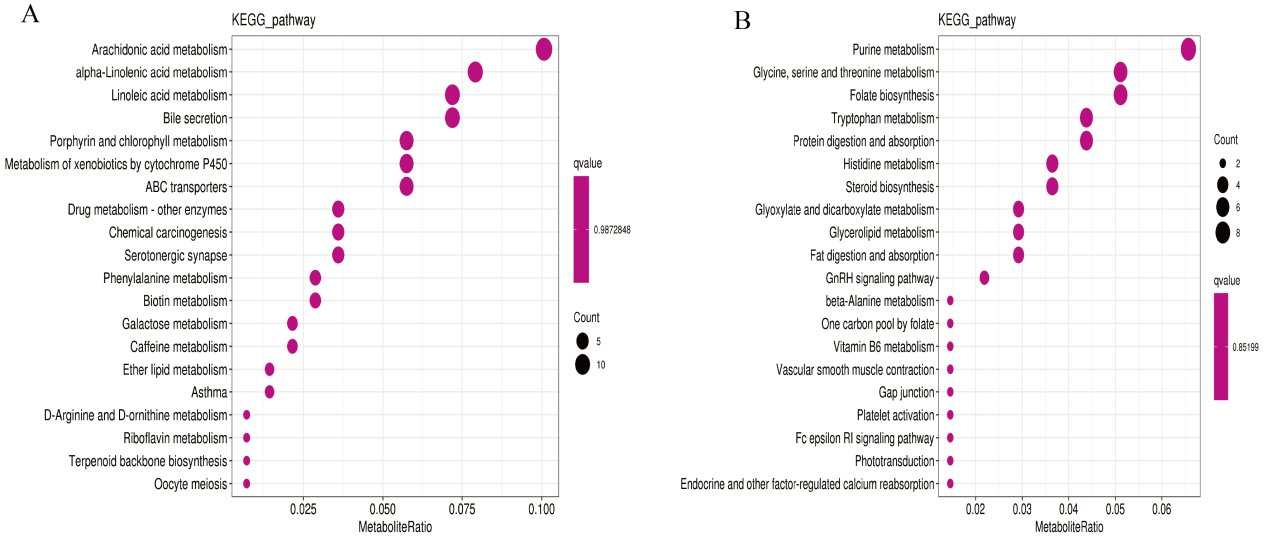
Fig S3 **a** KEGG pathway in positive model. **b** KEGG pathway in b negative model

**
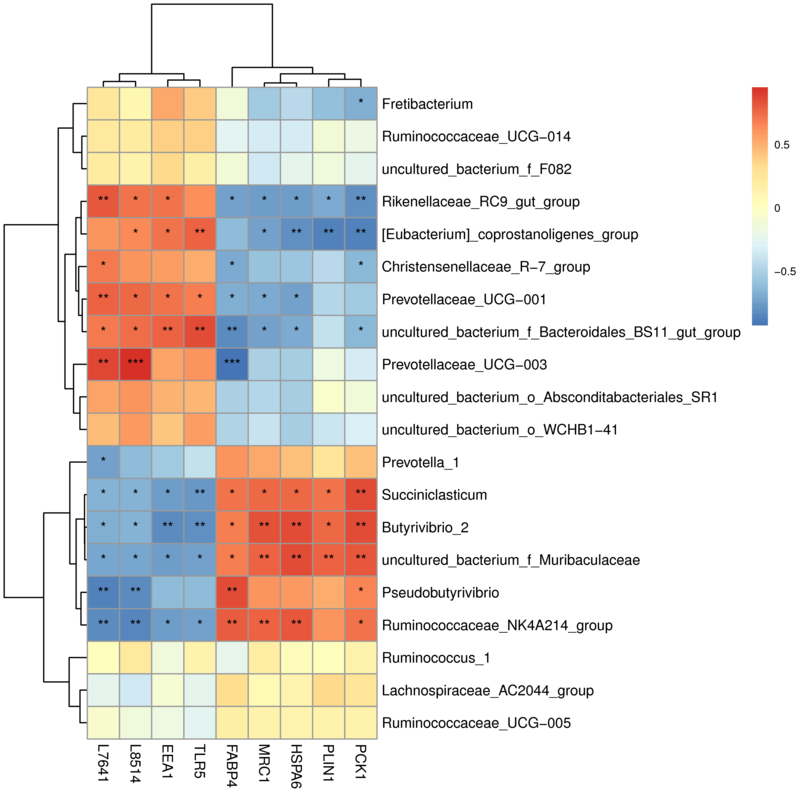
**

**Fig. S4** Correlation analysis between rumen microbes and host genes


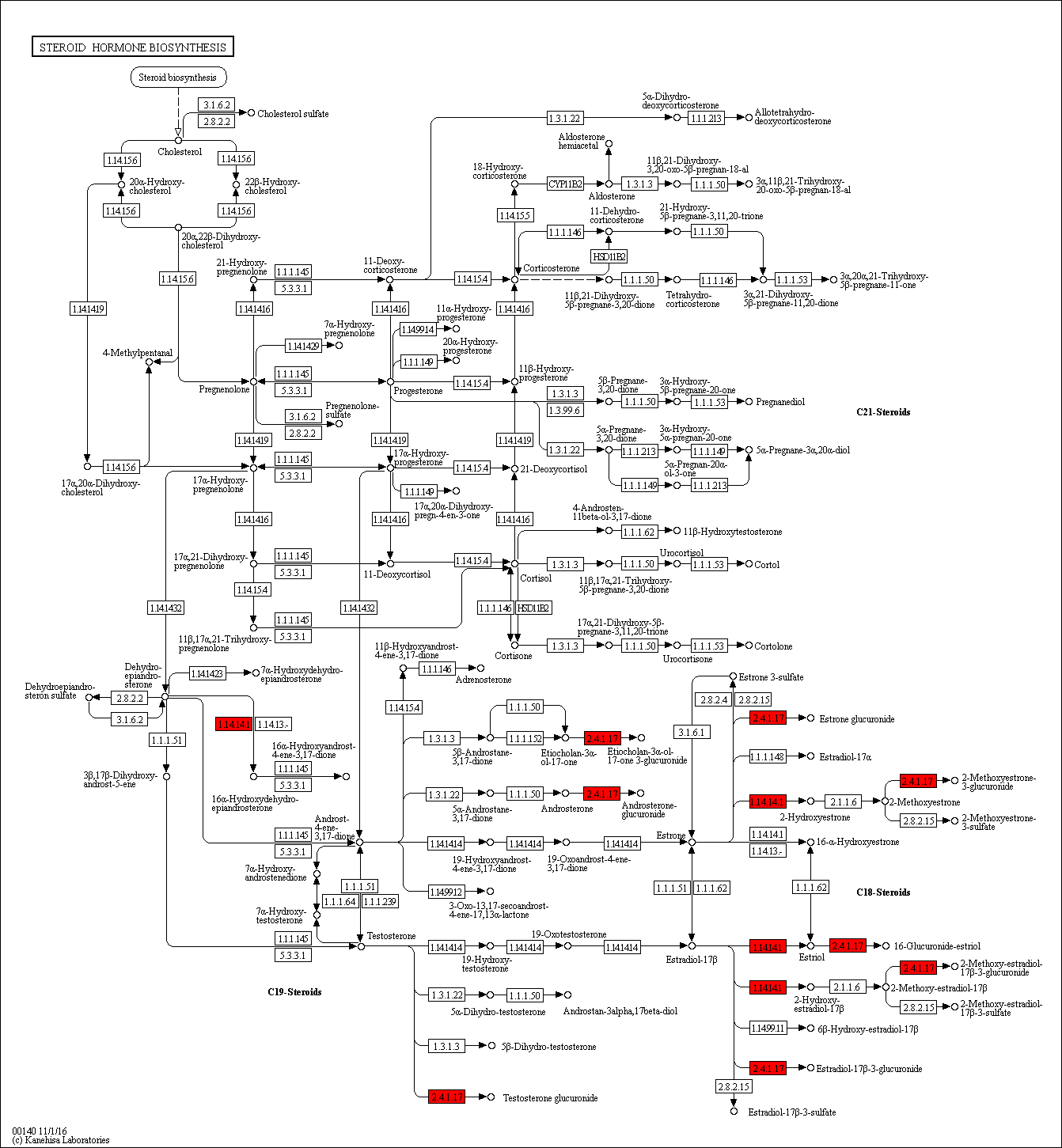


Fig S5 Ko00140 pathway
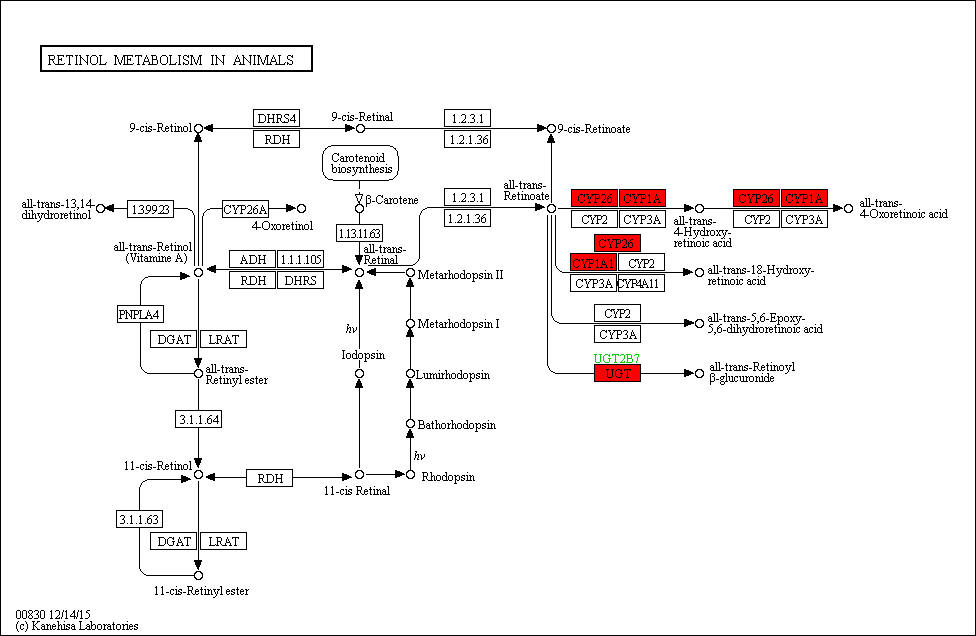


Fig S6 ko00830 pathway
